# Morbid Anatomy of the Liver

**Published:** 1837

**Authors:** S. D. Gross

**Affiliations:** Professor of General and Pathological Anatomy, in the Medical Department of the Cincinnati College


### Art. III.—Morbid Anatomy of the Liver.

*Outlines of the Morbid Anatomy of the Liver.* By S. D. Gross, M.D., Professor of General and Pathological Anatomy, in the Medical Department of the Cincinnati College.

That correct views may be entertained of the morbid changes of any organ, it is necessary that the student should have a thorough knowledge of the appearances which it presents in the normal state. Without a competent share of information of this kind, it is obvious that no physician, whatever may be his talents or acquirements in other respects, can successfully fulfil the duties of a pathological anatomist. So numerous, indeed, are the sources of fallacy, that even the most enlightened members of the profession sometimes fall into the most egregious blunders; and if this be the case, in regard to them, how much more likely must mistakes be to happen in the hands of the young and inexperienced, who have seldom ever been present, perhaps, at a necroscopic examination. That some are naturally better calculated to conduct such researches than others, cannot be denied; for there are those whose vision, both physical and mental, is so obtuse as to disqualify them forever from the task. But let no man deceive himself. There is no royal road to knowledge; and he who would become respectable, even as a morbid anatomist, must study; nor must he limit his attention, as is so often done, to the confines of the principal organs of the body. With scalpel in hand, he must penetrate every part, and bring to light every thing that nature has taken so much care to hide from his observation. Superficial examinations are worse than useless. From the tendency they have to lead to false deductions, errors of practice, often the most fatal, may be the result. Nowhere is the maxim "*whatever is worth doing should be done well*," more applicable than in the present instance.

In accordance with these principles, which should be inscribed upon every physician's conscience in letters of gold, I proceed to take up the consideration of the healthy structure of the liver, preparatory to entering upon that of its various lesions. The remarks I intend to offer, shall be as concise as may be properly consistent with the main object of this paper.

1.—The liver, situated, as every body knows, in the right hypochondriac region, together with a part of the epigastric and left hypochondriac, is a large glandular organ, from ten to twelve inches in breadth, from six to seven in length, and from four to five in thickness. In *weight*, it varies from two pounds and three-quarters to three pounds and a half; consequently, it may be said to constitute about the thirty-fifth part of the entire weight of the human frame. Its volume, however, it should be observed, is much influenced by the state of its own circulation; a circumstance which might be anticipated from the immense quantity of blood which is continually poured into it by the hepatic artery and portal vein. Of the extent of this influence, a good idea may be formed by removing the liver from the abdomen, and washing out its fluids; when its bulk will be found to be considerably diminished, as is shewn by its collapsed and wrinkled aspect. Nor is this all. Whatever has a tendency to retard, interrupt, or prevent the return of the blood from the hepatic veins to the right side of the heart, must induce congestion, with all its necessary evils, in the organ under consideration. Hence it is found that disease of the liver is often directly dependent upon lesion of the heart or large veins.

2.—That the *color* of the liver, the next subject to be noticed, should be modified, and, if I may so express myself, controlled, by the amount of blood which it receives, no one can for a moment doubt. Accordingly, we find, that, in the fœtus, in which the liver is always remarkably vascular, the color is much more florid than some time after birth, or in the subsequent periods of life. In the adult, it is generally of a reddish-brown, with spots of blue, particularly along the anterior margin and under surface. In persons who are hung, the liver is usually of a deep pink color; sometimes quite purple. Such, in a few words, is the healthy complexion of this important organ. How it is altered by disease, will appear in the sequel.

In simple *congestion*, the hepatic tissue is generally of a florid red; the arteries and veins are preternaturally distended, and blood flows freely on making sections of it. By a careless observer, this condition of the liver might be confounded with inflammation; but may be readily distinguished from it, by the greater uniformity of its color, by its pervading the entire organ, and by the facility with which the blood may be squeezed from its vessels. Dr. Kiernan, of London, has recently shewn, that when the congestion results from the portal veins, the granules are the parts which are most vascular; whilst, when it is caused by the hepatic veins, it is most distinct in the intervening cellular tissue. When both systems are filled with blood, the redness will be equally conspicuous in both textures. This is an important circumstance, and should not be lost sight of in our investigations.

3.—The *parenchymatous structure* of the liver is very fragile and lacerable, yet so compact that it cannot be much compressed. When torn, it assumes the appearance of small polyhedral granules, each of which may be considered as a perfect gland, as it is furnished with an appropriate excretory duct, together with nerves, blood-vessels, and absorbents. Their size equals that of a millet-seed, and their number is incalculably great. Lodged each in a distinct capsule of cellulo-fibrous substance, they are of a lighter color usually than the surface of the liver, and when a section is made of them, they appear to be porous; though whether this be really the case or not, is a point upon which anatomists are still undecided. Around them may be observed, in a collapsed state, the branches of the portal vein, accompanied by those of the hepatic artery, and those of the hepatic duct, the latter being frequently distinguishable by the exuding bile.

An important question here arises, not only in a physiological but also in a pathological point of view, namely: Are there two distinct sets of granules in the liver? Ferrein, Meckel, Andral, Hope, and other highly respectable anatomists, think that there are; and in support of their opinion, allege that in certain diseases of the organ, they are distinctly perceptible, one being yellow, the other red. Without entering into an elaborate discussion of the subject, which the limits of this paper would render improper, I shall content myself by observing, that, so far as my own researches go, I see no reason for adopting the sentiments of these eminent authorities. Nor am I singular in my want of orthodoxy upon this point. Professor Cruveilheir, of Paris, who may be considered as decidedly the best anatomist, both descriptive and morbid, of the present day, positively asserts, upon the evidence of a series of the most carefully conducted observations, that there is only one order of hepatic granules, lying in juxtaposition, and having each a cellulo-fibrous envelope.* The same sort of testi-

---

* Anatomie Descriptive, Tome ii. p. 567.

mony is borne by Dr. Kiernan, in his admirable monograph on the structure and functions of this organ, published in 1833. He states that he has satisfied himself by repeated injections, by examinations with the microscope, and by experiments on living animals, that the two species of granulations described by Andral and others, are perfectly identical, both in their texture and office; and he attempts to account for the error into which they have fallen, by refering the difference of appearance to a difference of congestion, the color of these bodies being red when the portal capillaries are engorged, but light yellow when there is distention of the minute branches of the hepatic veins.

In the liver of the hog, in which these granules are unusually distinct, and of a polyhedral form, I have repeatedly looked for these two orders of acini, but never, in a single specimen, have I been able to make them out. For this reason, therefore, as well as from the testimony of the French and English anatomists whose names have been quoted, I am warranted in the conclusion that they do not exist.

In the infant, the acini are much less distinct than in the adult. When a section is made, the parenchymatous substance seems to be much more homogeneous, and if torn, it does not exhibit such a well-marked granular aspect. Its consistence, also, is more tenacious, and the organ is much less liable to rupture from accident.

4.—The liver is enveloped by two *tunics*, which, although they are intimately connected, differ from each other widely in their structure, and in the purposes which they are destined to fulfil in the animal economy. Of these, the external, after covering the greater part of the surface of the organ, forms a number of folds, improperly called ligaments, which unite it to the diaphragm and wall of the abdomen. Derived from the peritonœum, it is perfectly transparent, smooth, and lubricated, for allowing the liver to move, without detriment, upon the surrounding parts. No vessels can be discerned in it in the healthy state; and when diseased, it often adheres to the neighboring structures by bands of adventitious cellular tissue.

The other membrane, usually named the *proper coat*, is much the more important of the two, inasmuch as it may be considered as constituting the frame-work of the entire organ. Not only does it enclose and sustain the liver, but sends thousands of processes into its interior, which, intersecting each other in every conceivable direction, form so many minute capsules for the hepatic granules. Thus, each of these little bodies may be viewed in the light of a representative of the entire gland, as it is not only furnished, as already stated, with appropriate vessels and nerves, but likewise with its own proper covering.

In structure, this tunic assimilates itself to the fibrous membranes. It is by no means so close and thick as the pericardium, with which, however, it has many important characters in common. Thus, for example, it is composed of fibres which interlace in different directions, and is in contact on one side with a serous membrane, which, in both cases, answers a similar purpose; that, namely, of facilitating the movements of the respective organs. Nor does the analogy cease here. In inflammation of the fibrous coat of the liver, the disease is generally propagated to the parenchymatous structure, in virtue of the processes which the membrane sends into the interior of the organ, and also of its close connexion with its outer surface, in the same manner as an inflammation of the pericardium may be propagated to the muscular substance of the heart along its serous investment.

Much difficulty is experienced, and much dexterity required, in dissecting this membrane from the part with which it lies in contact. On the concave surface of the liver, it is almost impossible to display it, but anteriorly, and especially towards the upper margin, it is very easy to raise it in the form of a strong, thick, dense, and consistent coat, inelastic, and of a light greyish color. In a peparation in the museum of the Cincinnati College, the membrane in question is nearly the twelfth of an inch in thickness. Superiorly it runs into the cellular tissue which is so abundantly found at the upper border of the liver, and which serves the important purpose of connecting the organ with the diaphragm. The mode of this connexion should never be lost sight of in our pathological investigations. By bearing it in mind, we shall be enabled to account for the manner in which disease may be propagated from one of these structures to the other, and how the matter of an abscess that is originally formed in the liver, may find its way into the lungs, and be finally expelled by coughing.

The discovery of this important tunic has been claimed by some of the German anatomists for Professor Sœmmering; by the French, for the celebrated Laennec; but the truth is, it was described more than a hundred years ago by Dr. Grieve, an English physician.*

5.—Nor can all the good be done by paying attention simply to the healthy and morbid appearances of the liver. To the practising physician, with a view of determining the diagnosis of some of the structural lesions of the organ, a knowledge of its *situation*, in respect to the neighboring parts, is of tantamount importance. Such information will be in the highest degree useful, also, as enabling him to comprehend how an enlarged liver may compress the stomach and interrupt its functions; how it may produce jaundice, by impeding the flow of bile; dropsy of the abdomen, by obstructing the return of the blood to the heart; how abscesses, originally

---

* De Secretione Bilis, Edinburgh, 1731.

formed in the hepatic tissue, may burst into tha surrounding organs.

The position of the liver is sometimes singularly altered, even although it may not have undergone any change in bulk. Thus, in hydro-thorax, it may be prominently felt, not only in the epigastric, where a part of it is naturally situated, but also in the umbilical, and left hypochondriac regions. On the other hand, in copious ascitic effusions, it may be forced so high up into the chest, as to encroach seriously upon the right lung, producing atrophy in that viscus, and terrible dyspnœa. Sometimes, again, the liver is pushed over into the left hypochondriac region, by an encysted tumor, or by enlargement of the right kidney. Now all these facts should be borne in mind by the physician, as they have an important relation to the diagnosis of abdominal diseases.

The *lesions* which will be treated of in the present essay, are, inflammation, suppuration, gangrene, softening, induration, hypertrophy and atrophy, tubercles, cancer, melanosis, hydatids, adipous degeneration, worms, cartilaginous and osseous concretions, apoplexy and laceration.

6.—*Inflammation* of the liver may involve either its proper substance or its investing membranes, and like the same disease in other organs of the body, it may be either acute or chronic. The acute form of the complaint is of rare occurrence in this country, but is sufficiently common in hot climates. In warm latitudes, there is always, as is well known, a high degree of nervous excitability of the chylopoietic viscera, and hence nothing is more usual than to see a large proportion of the maladies of those regions characterized by a predominance of inflammation of the stomach, liver, bowels or spleen. In our southern States almost all diseases assume a bilious type; but whether the liver labors under much inflammatory irritation, or is merely sympathetically affected, are points which still remain undetermined. The term *congestive*, which is so much in vogue with the physicians of those regions, seems to me to be often used unmeaningly, certainly without a proper regard to the fundamental principles of pathology. It is with them a sort of talisman, a mantle which covers a multitude of sins in the way of unsuccessful practice. That the internal organs, and especially the liver and spleen, are often inundated with blood in the intermittent and bilious fevers of those districts, may be readily supposed, but that this condition can last long without inflammation, is what very few will be willing to concede.

Nowhere, indeed, is there to be found a more striking instance of the operation of what Mr. John Hunter and his followers have denominated *continuous sympathy*, than in the viscera now under consideration. What takes place in the conjunctiva of the eye in inflammation of the Schneiderian membrane of the nose, in the fauces in inflammation of the larynx, or in the neck of the urinary bladder in inflammation of the urethra, may be supposed to occur, precisely, in the inner coat of the gall-bladder, common and hepatic ducts, in acute duodenitis, gastritis, or enteritis, or in these different affections conjoined. Here disease is propagated directly from one part to another, because of the similarity of structure of the lining membrane; nor is it uncommon to find that the organ which is secondarily involved, is obliged to bear the brunt of the irritation. In this manner are produced, there is every reason to suppose, many of the so-called cases of congestive fever of the south; the disease, in the first instance, being probably nothing but acute inflammation of the gastro-duodenal mucous membrane, which, by extension, implicates the mucous membrane of the liver and gall-bladder, and thus creates that obstruction in the portal circle so much spoken of by writers and practitioners.

True it is, the question has not yet been settled by post-mortem examinations, which are alone competent to do it; nevertheless, let any one reflect, coolly and dispassionately, upon the subject, and all the difficulties which would seem, at first sight, to environ it, will speedily vanish. Who does not know that the whole liver is lined by a mucous surface, the area of which, could it be properly estimated, would, in all probability, be found to be fully as great as that of the stomach, certainly as great as that of the duodenum, with which it is directly continuous? Should any one say that this is an exaggerated and over-wrought picture, let him contemplate, even if it be but for a moment, the large volume of this organ and its myriads of granules, and then take into consideration the fact, that each of these bodies, small as it is, is provided with an excretory tube, lined by a mucous membrane, so fine that even the most powerful microscope is scarcely able to detect it. Let him, we say, but do this, and all skepticism will at once fly from his mind. He will no longer regard the liver simply in the light of a parenchymatous structure, but as a viscus composed essentially of mucous follicles, subject to the same diseases as the duodenum which adjoins it, and which sends into its interior one of its most important tissues. Thus do we perceive how disease may be propagated from one of these organs to another; how an irritation seated in the mucous membrane of the duodenum may affect that of the liver; how the functions of the latter may be impaired by the former; and, conversely, how an inflammation of the liver may be transferred to the duodenum, the stomach, and small intestine, and commit ravages the most serious and extensive.

Did time permit, illustrations the most convincing might be adduced, in corroboration of the mutual dependency of these organs; but as we have already digressed so far, I shall bring this part of my subject to a close, by expressing the hope, that the practitioners of the southern States, many of whom justly rank amongst the brightest ornaments of the American profession, may be induced to bestow due attention on the anatomical characters of the various diseases which it is their province to treat. By diligent and carefully conducted necroscopic investigations, much light might be accumulated, that would be of the greatest value to the physician, as well as to the public at large.

7.—Inflammation of the liver appears occasionally to assume an epidemic type, affecting a considerable number of persons at the same time. An instance of this tendency occurred in 1828, in the city of Dublin, where, as we are informed by Professor Stokes, prior to this period, acute hepatitis was looked upon as a very rare disease; a case being seldom met with more than once in a year or two, in their largest hospitals. At the date here specified, however, every institution of the kind in the Irish metropolis had a considerable number of individuals afflicted with it, and of these not a few died.

8.—The anatomical characters of *acute hepatitis*, vary, as might be expected, according to the intensity of the irritative action, and the length of time it has been in operation. In general, the first perceptible effect is an increase of the vascularity of the parenchymatous structure, which becomes engorged in circumscribed spots, of a light red color. The part affected is somewhat tumid, abnormally dense, and bleeds freely on being incised; the hepatic canals are distended with a viscid, yellow, brownish fluid; and the acini are increased in size, and of a florid tint, though sometimes the reverse is the case, these bodies being much less distinct than natural.

In a more advanced stage, in addition to the vascularity, the hepatic structure becomes singularly softened, so that it may be reduced by the slightest pressure of the finger to a mere pultaceous mass. This state is analagous to the second stage of acute pneumonitis, and, like it, may be accompanied by the formation of pus, or a deposition of lymph upon the serous surface. In this respect, however, there is a wide difference between the liver and the lung, as we seldom meet with inflammation of the substance of the latter without pleuritis, while the reverse often obtains in acute hepatitis.

In its transition from the first to the second stage, the inflammation gives rise often to various shades of color. Thus, the parenchyma may be of a deep red, disposed in arborescent lines; or the vessels may encircle the acini in the form of wreaths, which freely anastomose with each other. When the disease is very intense, it is not unusual to see a number of small ecchymoses, produced by a rupture of some of the capillary arteries or veins; and instances frequently occur in which the affected part assumes a deep brownish, violet, or mottled aspect, the latter appearance being particularly liable to happen when the inflammation occurs in circumscribed spots. In such cases, the whole portal circle is gorged with black blood, the biliary ducts are deeply injected, and their contents being of a sero-sanguinolent nature, have no longer that bitter taste and ropy feel, which characterize healthy bile. The mucous coat of the duodenum, also, is more or less phlogosed, and the minute branches of the hepatic artery and portal vein no longer admit injecting matter.

It is not often that the whole of the liver is involved in acute inflammation; most commonly it is limited to its surfaces, to its borders, or some of its deep-seated parts. In the latter case, the disease sometimes escapes the attention of the examiner, owing to the normal aspect of the exterior of the organ. Hence the necessity, in all our investigations, after having tied the large vessels, of removing the liver from the abdomen, and cutting it into thin slices. In some cases, especially such as follow surgical operations, the hepatic and portal veins are inflamed; and cases occasionally occur, in which these tubes are filled with purulent matter.

9.—When the inflammation is seated in the investing membranes, they exhibit the same appearances pretty much as the fibro-serous textures generally. Numerous vessels pervade them, passing up from the parenchymatous texture, and they are thicker and more opaque than in the healthy state. Lymph is also commonly deposited upon their outer surface, either in narrow bands, in small patches, or in considerable masses. The organ being thus glued to the neighboring parts, is often compressed, and much restrained in its movements. In some instances it is literally buried in lymph, and so strong are its adhesions, that much dissection is required before it can be removed from its situation. The disease, however, is seldom thus extensive; in the generality of cases, it is limited; and, if I may judge from my own observations, it has a peculiar predilection for the anterior segment of the membranes.

Does disease of the substance of the liver ever exist without involving its coverings, and, conversely, of its coverings without involving its substance? Upon this point, different opinions have been expressed by different writers; some answering affirmatively, others negatively. M. Bonnet, a recent French writer, and evidently a disciple of the Broussais school, maintains that inflammation of the hepatic parenchyma is almost always accompanied with inflammation of its peritonæal investment. The arguments, however, upon which he endeavors to sustain his proposition have but a very slender foundation; and as they are directly at variance with the testimony of some of the best authorities of the present day, no further notice need be taken of them here. The dissections of Annesley, of Andral, and of Louis, are sufficient to negative a thousand speculative notions; and if any reliance is to be placed upon them, it must be clear enough to every candid mind, that the very reverse of the position assumed by M. Bonnet, is the true one. Not only do these pathologists assert that hepatic peritonitis is of extreme rarity, but they declare, upon the evidence of repeated personal observations, that the substance of the organ may be greatly altered, nay, even seriously disorganized, and yet its tunics be perfectly sound. "Even abscesses of the liver," says Mr. Annesley, "may proceed to the utmost extent, and ultimately break into the abdominal cavity, without having induced inflammation of the serous surface, where they point, and consequently without forming adhesions to the parts with which they are in immediate and close contact."

10.—Not much need be said here of the *symptoms* of acute hepatitis, as all the information that the student may desire upon this point can be obtained in the common treatises on the practice of medicine. The local phenomena, to which my remarks will be limited, may be referred principally to the pain, tenderness, and swelling in the region of the affected organ. When the disease is seated in the parenchymatous structure, the pain is of a dull, heavy, aching character; if in the investing membranes, it is sharp, lancinating, and often extremely severe. In either case, but especially in the latter, it is *constantly* increased by pressure, by inspiration, and by coughing. In some instances, the pain is felt in the right shoulder, along the spinal column, under the short ribs, in the loins, or in the epigastric region; but as these symptoms may arise from other affections, as, for example, inflammation of the top of the right lung, incipient phthisis, pleuritis, aneurism of the right subclavian, gastritis, colonitis, or disorder of the kidney, they are perfectly worthless as diagnostics. The patient lies commonly on the affected side, seldom on the back or on the left side; in which respect there is a striking difference between this disease and acute pleuritis, in which the person always lies on the sound side.

The tumefaction of the liver, which, next to the pain and tenderness, may be regarded as the most important symptom, is seldom considerable. When it is, the right hypochondriac region appears unnaturally full, and the margin of the organ can be felt beneath the edges of the false ribs. In some instances, the costal cartilages seem to be tilted out; but in such cases, the intercostal spaces retain, almost invariably, their depressed appearance; affording thus a striking contrast between hepatic and pulmonic disease, in the latter of which, when the cavity is distended, the intercostal spaces are either flat, or pushed beyond the level of the ribs.

Such then, are the three signs upon which our principal reliance is to be placed, when endeavoring to make up our diagnosis of acute hepatitis, *pain, tenderness on pressure, and swelling*. Add to these, the position which the patient assumes in bed, the derangement of the digestive organs, and the inflammatory state of the system, and little or no difficulty can occur in coming to a correct decision.

11.—Acute inflammation sometimes terminates by resolution, but much oftener by the formation of matter, gangrene, or softening of the hepatic texture. Not unfrequently it passes into the chronic form, when it is liable to give rise to induration and other structural lesions. There is reason to believe that chronic hepatitis sometimes exists as a primitive affection, its invasion being very gradual, and unaccompanied by any severe symptoms. It is a much more frequent complaint in this country, than the acute form; yet it is not quite so common, perhaps, as the term "liver-disease," so generally used by the profession, would lead us to suppose. The anatomical characters of the disease, together with its diagnostic symptoms, so far as they are understood, may be collected from what we shall say concerning induration, hypertrophy, atrophy, and fatty degeneration of this organ.

12.—Although *suppuration* of the liver occasionally follows chronic inflammation, yet this effect is much more frequently witnessed as the result of the acute variety of the disease. The pus may be diffused, occur in small points, or be collected into one or more abscesses. The number of these purulent deposits is often quite great, the liver being literally burrowed by them; sometimes they communicate together by narrow fistulous passages, but in most cases they are separated by considerable intervals, or by thin partitions of hepatic substance. Occasionally the abscess is of extraordinary size, occupying nearly the whole of one lobe, or the greater part of the organ. In this way quarts of matter are sometimes accumulated. When the quantity of fluid is great, or if it be long detained even when small, it is apt to become encysted, the membrane by which it is enclosed, and which is susceptible of organization, varying in thickness from the fourth of a line to half, an inch, and in consistence from the softness of gristle to the firmness of fibro-cartilage. In other cases, especially in such as run a very rapid course, the matter lies in immediate contact with the hepatic tissue, which is itself usually in a state of purulent infiltration, or very much softened and altered in color.

13.—The matter of a hepatic abscess usually partakes a good deal of the nature of true pus, being of a thick, white, cream-like appearance. Sometimes it is thin and sanious, sometimes thick and curdy, sometimes thin and brownish like chocolate, and sometimes of the color and consistence of a decoction of unburnt coffee. In the generality of cases, it is destitute of smell; but occasionally it is highly offensive, and irritating. Now and then the fluid contains flakes of blood of a dark grey color; and instances occur in which it looks like the washings of flesh.

13.—Suppuration of the liver does not always prove fatal. In many instances the matter has a tendency to discharge externally, or to force its way into some one of the adjacent organs. The principal circumstances which appear to influence the point of communication are the seat of the abscess and the general volume of the hepatic tumor. In the abdomen, the opening usually takes place in the peritonæal sac, the colon, duodenum, the stomach or gall-bladder; when the abscess points externally, the matter may find its way out at the epigastric region, the right loin, or between the false ribs; and in either case it generally pursues a narrow fistulous route. It is not improbable that the pus formed in the substance of the liver, may sometimes pass into the bowels through the biliary ducts; and authors mention instances in which it found an outlet through the right kidney. When the abscess is seated at the upper border or on the convex surface of the liver, it not unfrequently breaks into the right side of the chest, where it either excites fatal inflammation, or erodes the pulmonary tissue, and is finally discharged through the bronchial tubes between the ribs, or even through the axilla. But the most extraordinary circumstance connected with this subject, is, that the fluid sometimes works its way into the pericardium. Of this an interesting case has been reported by Dr. Smith. The liver adhered closely to the upper part of the diaphragm, and was occupied by an enormous quantity of pus, about a quart of which had escaped into the pericardium. When the matter is discharged, the abscess sometimes heals, the process being analagous to that which is observed under like circumstances in the lungs and other organs.

Hepatic abscesses, on the whole, I have reason to believe, are very rare in this country, having never, in all my dissections, seen more than two cases. In Europe they are also extremely infrequent;* but they are sufficiently common in hot climates, and nowhere, perhaps, more so than in the East Indies.

In acute hepatitis, pus usually begins to be deposited in the course of six or eight days from the commencement of the attack; whereas in chronic cases months often elapse before the occurrence of this event. When suppuration is about to take place, there is a sudden aggravation of all the symptoms; the patient experiences frequent rigors, followed by profuse sweats; the pain in the side is of a throbbing, lancinating nature; and there is a peculiar sense of sinking, with anxiety and precordial oppression; the organ is commonly somewhat enlarged; and although there is no distinct fluctuation, especially if the matter be deep-seated, there is almost always a boggy, yielding sensation imparted to the finger when passed over the region of the tumor. This sensation, as might be anticipated, will be more distinct in proportion as the purulent fluid approaches the surface, and in this case, too, the

* Andral's Path. Anatomy, vol. 11, p. 373.

diagnosis will be much aided by percussion. In the natural state, the hepatic tissue is so dense as to afford a very dull sound when we strike the hypochondriac region; but in superficial abscesses, especially in such as do not contain too thick and consistent a pus, there is usually a considerable degree of resonance, which serves to define the limits between the healthy aud diseased parts. After all, however, the nature of the lesion is often extremely obscure, and it may be truly said that there are few occasions for finer diagnosis than in the affection in question.

Abscesses of the liver are liable to be produced by irritation of the lungs; and in many instances they supervene upon injuries of the head, large wounds, and surgical operations. The matter in these cases is supposed by some to be transmitted with the blood through the veins, from remote and diseased parts; but the more philosophical opinion, and that which meets my own decided approbation, ascribes its origin to inflammatory irritation of the substance of the liver, brought on by some inscrutable sympathetic action. That matter may be thus conveyed, no one will deny; but that it should create irritation in one organ in preference to another, is a point in pathology which cannot be very easily comprehended. If it has any truth at all, it forms merely an exception to the general rule. To admit that abscesses may be thus produced in any organ, is to admit that such an organ has an elective attraction for the purulent fluid that shall be flowing in the circulating mass; or, on the other hand, that its tissues are so close and peculiar, its capillaries so exceedingly fine, as to entangle the matter, and retain it, *vi et armis*, as it were, in its interior. To me the hypothesis appears not only objectionable, but too absurd almost for serious consideration.

14.—The termination of acute hepatitis by *gangrene* is so rare, that many writers have denied the possibility of its occurrence. Baillie never witnessed an instance of it,* and Annesley, although for many years an inter-tropical practitioner, and constantly in the habit of making examinations, declares that he has not been more successful.† In several thousand dissections made by Andral,‡ this lesion occurred only in a single case. From all these facts, then, it may be justly concluded that gangrene of the liver is of great rarity. Portal describes the mortified part as being extremely offensive, of a dark color, pervaded with sanious fluid, very soft and lacerable. In a case recently detailed by Dr. Stokes, of Dublin, the organ bore numerous marks of chronic disease, and in the left lobe was a cavity which was distinctly gangrenous, having

---

* Morbid Anatomy, p. 136.

† iseases of India, vol. 1, p. 435.

‡ Path. Anatomy, vol. 11.

in its centre a large mass of slough. The patient, an old drunkard, complained during the last few days of his life, of great pain and tenderness of the abdomen; and a short time before he expired he was seized with vomiting, and threw up a large quantity of fœtid matter.

15.—*Softening* of the liver is said to be a very common and destructive affection in warm latitudes; but it is very rare, I presume, in the more temperate regions of America and Europe. That this is the case I am disposed to believe from my own experience, and from the few cases that are reported of this disease in our periodicals. Softening of the liver presents itself under two distinct forms, differing from each other both in their color and consistence, but resulting probably from the same cause, namely, inflammatory irritation. In the more common variety, which I shall denominate *brownish softening*, the substance of the organ usually retains its normal color, but is broken down and friable, or has so far lost its cohesive properties that it readily yields to the force of the finger. In some instances the affected part is infiltrated with thin, bloody, sanious, or puriform matter, and is converted into a soft, brittle mass, not unlike a rotten pear. When put in water, an immense number of small granules appear, resembling the seeds of a bunch of dried grapes; they are of a yellowish, brownish or cinnamon color, and are attached to the large vessels by delicate vascular pedicles. In other cases, maceration produces an appearance like the washings of half-putrid flesh, the water becoming discolored, and the part exhibiting multitudes of shreds, the remains probably of vessels and fibrous filaments. This variety of mollescence is generally most remarkable on the convex surface of the liver, and occurs almost always in combination with diseases of the other viscera. especially of the stomach, spleen, and lungs. According to Mr. Annesley, it is frequently met with in India, in persons who are rapidly carried off by cholera, dysentery, and malignant fevers.

16.—The *second species* of softening has received the name of *black*, and, from its close resemblance to gangrene, is probably the result of a higher grade of inflammatory action, disorganizing the parenchymatous texture of the liver. It commonly invades a large extent of surface, sometimes indeed almost the whole organ, which it reduces to a dark pultaceous mass; having the consistence and aspect of grumous blood, or of a black, softened spleen. In some instances the affected part emits a remarkably fœtid odor; a circumstance which, together with its color, has given rise to the belief that the disease is analagous to, if not identical with, gangrene. The size of the liver is commonly altered; in most of the cases of which we have any account, it was reduced to one half or less of its normal volume; it is occasionally, I presume, enlarged, but this does not occur often.

Very often there are no symptoms which denote the existence of this fatal lesion during life. In a case recently communicated by Dr. C. A. Lee, of New York, in which the liver was completely disorganized, and converted into a black mass, like grumous blood, the patient, a colored man, of intemperate habits, forty years old, suffered no other inconvenience than diarrhœa and slight debility; he was not confined to his house, and suddenly expired while walking the street.* In another case, mentioned by Dr. Abercrombie,† the symptoms were jaundice, want of appetite, and nausea, followed towards the last by vomiting of black matter: there was no pain, no tenderness, no fulness in the region of the liver. From these and various other facts of a like character, it may be inferred, that the disease, commencing in the parenchymatous structure of the organ, progresses in a stealthy and insidious manner in its work of disorganization, without always particularly impairing the general health.

Neither Morgagni, nor Haller, nor Baillie seem to have ever met with this disease; and although numerous examples have been narrated by more recent writers, a good description of its anatomical characters is still a desideratum. The causes of this singular lesion are not well understood; but that it depends upon inflammation, either acute or chronic, is an opinion which seems to be borne out by an attentive examination of the various cases of it which are to be found upon record.

17.—There is a state of the liver, not at all unusual, the very reverse of that which has been now described. I need scarcely say that I refer to *induration.* In this disease, the substance of the organ is unnaturally hard, firm, and dense, exhibiting, when torn, a singularly granulated surface. The volume of the gland is sometimes unaltered, but in most cases it is much reduced, occasionally nearly one half. The color varies in different instances, being either a pale red, purple, nutmeg, brown, or lilac. Cruveilheir informs us that he has often seen induration of the liver accompanied with a green olive hue, but of this I have never witnessed an instance; nor am I aware that the same observation has been made by other pathologists. This change, which I am inclined to believe commonly results from chronic inflammation, is often characterized by great thickening of the fibrous structure, and by a remarkable atrophy of the hepatic granules. In some cases, indeed, the granules seem to be entirely removed, their place being occupied by a cellular tissue, very hard, and of a dull greyish color. The tunics of the liver usually participate in the condensation; the cellular substance accompanying the ramifications of the

---

* American Journal of the Medical Sciences, vol. 17, p. 60.

† Pathological and Practical Researches, p. 357.

hepatic artery and portal vein is frequently indurated; and many of the excretory ducts are permanently obliterated, whilst others are thickened, and of almost gristly consistence.

The induration is not always partial; I have frequently seen it pervade the entire organ, though in the majority of cases it occurs in patches, or is limited to particular parts. The right lobe and lobule of Spigelius are perhaps more generally implicated than any other portions. The disease is often attended with pain in the hypochondriac region; the countenance is wan and sallow; the conjunctiva is more or less yellow; and the patient is liable to be affected with ascites. The secretion of bile is frequently very sparingly performed, and the properties of this fluid are singularly altered. These symptoms, however, are far from being constant; nor can they be considered, when present, as diagnostic of hepatic induration.

18.—*Hypertrophy* of the liver is a frequent affection. This state is said occasionally to exist simply, but the assertion, I think, may well be doubted. In by far the greater number of cases, if indeed not always, it is combined with one or other of the states which we have just described, induration, and softening. The color, size, and form of the liver in this affection vary infinitely according to the period of its existence and the nature of the primary lesion. The most common tints are pale red, grey, brownish, dark green, mahogany, and nutmeg. Frequently these colors are more or less blended, so as to impart a variegated appearance to the parenchymatous structure.

The bulk of the organ is sometimes enormous. Cases have been recorded in which the human liver weighed from twenty to thirty pounds; and in animals, as the horse and ox, the weight not unfrequently exceeds one hundred pounds. In a case mentioned by Dr. Ruyer in the Encyclographie des Sciences Medicales for 1835, the organ weighed twenty-four pounds.* It was taken from a woman thirty-three years of age, was of a brownish slate color, soft and friable, and filled the whole abdominal cavity, forcing the diaphragm high up into the thorax, and pressing the bowels and other viscera firmly against the spinal column. In its substance were contained numerous whitish tumors, varying in size from that of a mustard seed to that of a hen's egg, some of which were occupied by purulent fluid.

The hypertrophy may be limited to a single lobe, or it may occur in all of them at the same time; most commonly, however, it attacks the right side. The shape of the liver, as has been

*"Mr. Gooch gives a case in which, during dropsy, the liver acquired the monstrous weight of twenty eight pounds. Baldinger reports another instance in which it reached twenty pounds; and Bonetus a third, in which it weighed only two pounds less."

already hinted, may be variously affected; and the surface of the organ is remarkably rough, granulated, furrowed, notched, or mammillated.

Hypertrophy is most common in young strumous subjects, or in such whose constitution has been tainted by syphilis, and is usually connected with a low and protracted inflammatory state of the parenchymatous structure. The affection is rarely attended with much inconvenience, save what arises from the increased bulk of the organ. In the generality of instances, there are jaundice, dropsy of the abdomen, irritable stomach, and constipation of the bowels, with enlargement of the spleen, and a sense of weight in the right hypochondriac region.

19.—*Atrophy* of the liver is much less frequent than hypertrophy, and, like it, usually occurs in combination with induration or mollescence. I have seen it repeatedly affect the whole organ; but it is much more common to find it limited to one of its lobes; and what is rather paradoxical, the liver may not only be of its natural dimensions, but even enlarged. In speaking of induration, mention was made of the fact that the granular structure is sometimes entirely absorbed, and replaced by dense cellular substance. In this case, although there may be no diminution of bulk, there is yet an actual wasting of the hepatic tissue, the part affected being reduced, as it were, to its primordial frame-work. The color of an atrophied liver presents every variety of shade, from greyish white to almost entire black.

This state of the liver is often observed in persons who are in the constant habit of using ardent spirits. It is rarely seen before the middle term of life, and is most common in old age. The hepatic vessels are always much diminished in size, and the functions of the organ more or less impaired. Its cause is manifestly chronic inflammation, producing an effusion of lymph and a partial absorption of the granular tissue. This atrophy of the liver is frequently connected with ascites, either as cause or effect; as was pointed out several years ago by Dr. Andral, and more recently by Dr. Seymour, in his valuable work on the nature and treatment of dropsy.

20.—As intimately connected with the two changes last described, it will be proper here to notice that peculiar state of the liver to which some of the French and British pathologists have applied the epithets *granulated*, *mammillated* and *tuberculated.* The affection seems to have first attracted the attention of Laennec, who has described it under the name of *cirrhosis*, in reference to the tawny yellow color which it occasionally presents. It was soon after noticed by Dr. Baillie, of London; and since his time, it has been particularly investigated by Andral, Bouillaud, Cruveilheir, Hope, Carswell, and other anatomists. The lesion seems to consist essentially in a hypertrophy of the hepatic granules, brought about by chronic irritation, acting in a slow and insidious manner. Their texture is dense and compact, and exhibits a great variety of tints, being sometimes of a deep brownish red, sometimes of a tawny yellow, like bees-wax, and not unfrequently of a pale cinnamon, pink, or lilac hue. When cut, they present a perfectly flat surface; and if further examined, are found to be invested by a dense cellulo-fibrous capsule, of a light greyish color, from which they can be easily removed by dissection, their principal bond of connexion being a small peduncle through which they receive their supply of blood-vessels, nerves, and absorbents. The size and shape of the granulations are variable. I have seen them of the magnitude of a common cherry; but usually they do not exceed a garden-pea, and often they are not larger than a mustard seed or a currant. Although mostly spherical, they are sometimes irregularly round, and even angular.

When these granules are very numerous, large, and of a variegated brownish color, they give the liver a peculiar *nutmeg-like* appearance. This state of the organ is said by Dr. Hope to be most prevalent in old dram-drinkers; but it may also result from repeated attacks of chronic irritation, or from long-continued congestion.* Hence obstruction of the vena cava or hepatic veins seldom exists long without producing enlargement of this kind.

In whatever form this species of hypertrophy occurs, the surface of the liver generally presents a singular mammillated aspect, as if it were raised into a multitude of spherical eminences; the peritoneal coat is thickened, opaque and wrinkled; and the volume of the organ is either diminished or augmented, seldom natural. In the former case, the hepatic texture is generally very dense, and almost destitute of moisture; in the latter, it is usually somewhat soft, more or less vascular, and impregnated with thin, bloody matter, which can be easily squeezed out with the fingers. In both varieties, but more especially in the first, the hepatic tissue is often of a yellow tinge; and the bile being retained in the rootlets of the hepatic duct, exudes pretty freely on making sections of it. If, in this state, a portion of liver be macerated for ten or twelve days in water, the little granules will assume the appearance of adipocire, and may be easily washed out of their cellulo-fibrous capsules, leaving them in a soft, flocculent condition.

21.—*Tubercles* are of very common occurrence in the liver, being either seated in its substance, or scattered over its surface; giving it, in the latter case, a very rough appearance. Lying in close contact with each other, or separated, as the case may be, by large intervals, these bodies are generally of a rounded shape, and consist of an opaque yellowish substance, in various degrees of

---

*Principles of Morbid Anatomy, p. 103.

maturation. Their size, although sometimes not exceeding that of a mustard seed, is usually considerable, many of them being as big as a cherry or even a hazel nut. When the liver is thus tuberculated, it feels much harder than natural, and its vessels are compressed and sometimes greatly reduced in volume, though the organ itself rarely deviates much from the ordinary standard. In some cases, the tubercles bear a strong resemblance to those of the lungs; they have the same size, the same form, the same consistence, and, in all probability, the same origin; but are a little browner in their color. They are obviously connected, in most instances, with a scrofulous diathesis of the system, as is manifested by their frequent co-existence with pulmonary phthisis, and by their contents being usually of a curdy nature.

Besides this variety of tubercles, Baillie has described another, which he has designated by the phrase, large white tubercle of the liver. His division, however, has not been generally adopted by succeeding writers, and may justly be rejected on pathological grounds, it being now pretty well ascertained that the bodies in question are merely a species of carcinoma, presently to be described.

Tubercles of the liver produce marked symptoms only when they are numerous, or when they are associated with hypertrophy or structural lesion. Under opposite circumstances they seldom cause much local inconvenience, or impairment of the general health. They are seen occasionally in old subjects, but are most frequent by far in children. Cruveilheir asserts that he has never met with them in adults who died of pulmonary phthisis, although he has made numerous examinations.* Louis, however, has been more successful; for in one hundred and twenty three cases of consumption, he met with these tubercles twice in the liver.†

I have also, in a few cases, found them in association with tubercular phthisis, though more commonly without it. On the whole, I am induced to believe that this lesion of the liver is of rare occurrence in this country. In some of the inferior animals they are sufficiently common, and in none more so than in the sheep, in which they are observable at a very early age.

22.—The liver is extremely liable to be affected with *cancer;* more so, according to some writers, than any other organ in the whole body. That this may be the case in some countries is probable, but that it is not in the United States I am convinced, both from my own observations, and from those of the profession at large. Be this as it may, cancer is always a most formidable disease, perfectly uncontrollable by medicine, and consequently inva-

* Dict. de Medicine et de Chirurg, t. viii. p. 329.

† Pathological Researches on Phthisis.

riably fatal. Two varieties of cancer are usually recognized by authors, namely, the carcinomatous, and the encephaloid.

23.—*Carcinoma* always occurs in distinct masses, the size, form, and consistence of which exhibit numerous diversities. The smallest are frequently not larger than a mustard seed, but in most cases their volume equals that of a chesnut, a walnut, or an orange. In a specimen in the museum of the Cincinnati College, the tumors, two in number, are about the size of a common apple; they are of a spherical form, hard, dense, and gristly, and situated in the very centre of the organ. The masses sometimes lie close together, and by their agglomeration form a tumor equal in size to a fœtal head; but such cases are rare, and I have never met with them. These heterologous structures seldom occur singly. In the majority of cases there are not less than six or eight, and instances have been known where there were as many as two or three hundred at the same time.

In their form these tumors are generally spherical; and those which project beyond the surface of the liver, for which they have a great predilection, have often a white greyish dimple in their centre, produced, it is supposed, by a condensation of the subserous cellular substance. The smallest, and those that are of recent formation, are of an opaque white appearance, and intimately connected with the surrounding tissues; whilst such as are old, and of great magnitude, are often of a brownish color, with various shades of grey and black, and can be easily lifted from their situation. Vessels frequently shoot into them, sometimes so large that they can be filled with injecting matter; nerves and absorbents, they also no doubt receive, but their existence has not yet been demonstrated by actual observation. To the touch they are hard and firm; and to the scalpel they offer the resistance of fibro-cartilage, the largest and densest yielding a slight creaking sound. The section generally displays a fibrous structure, the fibres radiating irregularly from the centre towards the circumference, like those of a turnip. In other cases, the tumor is lobulated and internally areolar, filaments of a greyish, brownish, or yellowish hue intersecting it in every possible direction. A juice may be obtained from them by pressure, and, in the cellular variety, a substance of the consistence and color of thin putty, paste, or concrete lard. Small masses of blood are sometimes diffused through them, but this occurrence is seldom witnessed, except in such as are old, or in a declining state.

The hepatic substance in the immediate neighborhood of these bodies, is, in most cases, perfectly sound. In others it is indurated, softened, infiltrated with different kinds of matter, or partially absorbed. Hypertrophy of the liver is sometimes observed; but a much more common occurrencc is an extreme reduction of its weight and bulk. When the tumors are numerous, the larger vessels are apt to be compressed, and, as a consequence, the organ is often exsanguine. From the same cause may result obstructions in the hepatic duct, with retention of the bile, and yellowish discoloration of the parenchymatous texture.

24.—*Encephaloid* disease of the liver is occasionally observed, though not so often as carcinoma, with which it probably has a similar origin. Like the preceding variety, this species of cancer occurs in various sized masses, of a greyish white color, soft, compressible, and elastic, with trifling vascularity. They offer very little resistance to the knife, and are composed of an areolar meshwork, occupied by a soft, pulpy, brain-like substance, intermixed with fibrinous concretions and dark-colored clots. The intersections are commonly of a lighter tissue, and, instead of being dense and thick, are eminently lax and delicate. Occasionally, though very rarely, the contents of these tumors are of the nature of hard jelly, constituting the *gelatiniform sarcoma* of Portal, Cruveilheir, and other pathological anatomists. The liver is sometimes much enlarged, and presents a mixed mass of disease, carcinomatous, encephaloid, melanotic, and tubercular, in various stages of maturation. The encephaloid matter is sometimes deposited in the portal and hepatic veins, as has been so ably represented by Carswell, in his Illustrations of Morbid Anatomy.

The symptoms of cancer of the liver are often exceedingly obscure, and frequently a long time elapses before the patient experiences much constitutional disturbance or local suffering. The disease in some instances appears to be latent, or manifests itself only a short time before death. The appetite is generally more or less impaired, the skin is sallow, the strength gradually declines, the body becomes emaciated, and there are frequent attacks of pain in the right hypochondriac region, with occasional nausea and vomiting. When the liver is much enlarged, and the cancerous tumors have attained considerable magnitude, the surface of the organ may impart a hard, nodulated, or lumpy feel to the hand applied over the abdomen, and this constitutes the only pathognomonic sign of which we have at present any knowledge.

25.—It is seldom that the liver is affected with *melanosis*. When it occurs here, it exhibits the same characters as in the other organs, and need not, therefore, be particularly described. I have never seen this disease in the human liver, but several times in that of the ox, in which it is by no means uncommon, especially in such as are raised in Ohio. In one specimen, the organ contained a great number of melanotic masses, arranged in the form of encysted tumors, varying in size from a small pea to a walnut. In man, the matter either assumes the tuberiform arrangement, or is diffused throughout the parenchymatous structure as an infiltration.

26.—*Hydatids* are of frequent occurrence in the liver, either occupying its substance, or studding its surfaces. Like tubercles, they may occur at any period of life, although they are witnessed most usually in adults, and persons who have been addicted to intemperance in eating and drinking. Commonly of a globular shape, they are often closely clustered together, and are almost always contained in a cyst, formed of a dense fibrous texture, of a white, opaque appearance. In an examination which I made about eighteen months ago, the hydatids looked like spherical bags, the largest of which was about the size of an onion, whilst the smallest was scarcely as large as a rifle-ball; their external tunic was dense, opaque, marked with numerous yellowish dots, and liberally supplied with vessels; their number did not exceed fifteen. The liver was much reduced in size, pale in its color, and its texture in many places so soft as to bc unable to withstand the slightest pressure of the finger.

These parasitic beings are not peculiar to the human liver; they occur also in that of animals, especially in oxen, hogs, and sheep. In these quadrupeds, the organ is sometimes completely crowded with them, immense numbers being every where scattered through its substance. In the liver of a cow, Mr. Youatt found nearly three hundred of these singular animals, varying from the size of a sparrow's egg to that of a swan. Some of the cysts were filled with blood, some with lymph, and many of them were intersected by fibrous bands. The liver in which they occurred weighed one hundred and thirty-seven pounds, and measured from one lobe to the other more than a yard and a quarter; its centre was perfectly fibro-cartilaginous, without any trace of its original structure.* This case is an exceedingly interesting one, and I mention it here not only with a view of showing the great number of hydatids that are sometimes congregated together, but also for the purpose of pointing out the fact, that the liver may be sometimes almost totally disorganized, and still the animal not evince any serious indisposition. The disease in this instance had no doubt existed for a long period, yet the cow's health was but little impaired; enlargement of the belly, yellowness of the skin, and slight emaciation, being the only symptoms which she exhibited previously to being killed by the butchers. In the human subject there are no manifestations which mark the presence of these bodies, distinct from those of chronic hepatitis. The disease may simulate ascites; and not a few cases are recorded in which the patient was actually tapped for this complaint.*

---

* The Management and Diseases of British Cattle, p. 460. Phila. 1836.

† See Medico-Chirurgical Review for March 1824. p. 930.

27.—Less distinctly organized are those *serous cysts* which are sometimes observed on the surface of the liver; constituting the disease which some writers have described under the name of encysted dropsy. The walls of these vesicles are generally transparent, smooth and polished, and supplied with numerous vessels, of the finest texture and most beautiful arrangement. Their contents, which are thin and limpid, like the water of ascites, are coagulable by heat, alcohol and acids. In the only case in which I have observed these tumors, they hung pendulous from the convex surface of the liver, were somewhat globular in their shape, and about the size of a walnut. Their most common size is that of a grape, but they may increase to a magnitude capable of holding many pints. Thus in a man aged thirty-two, Dr. Abercrombie found a cyst which contained eighteen pounds of fluid, and the walls of which were firm and dense, like thickened peritoneum. At the bottom of the sac were two coiled up cylindrical bodies, of a soft gelatinous consistence, which, when unrolled, had the appearance of false membranes, being about ten inches in diameter, and the eighth of an inch in thickness. The liver was not altered in structure, and all the other abdominal viscera were in a normal state. The patient had experienced much suffering, caused mainly, perhaps, by the encroachment of the immense tumor upon the diaphragm and surrounding organs.

28.—The liver has occasionally a peculiar *adipous* appearance, the fatty matter being either diffused through its parenchymatous structure, or confined to particular spots. The affected part is of a pale yellowish color, not unlike that of a pale autumnal leaf, its consistence is more or less diminished, it perceptibly greases the scalpel, is unctuous to the touch, and yields an oily principle by boiling. This, according to the recent experiments of Mr. Bird of London, is a soft brownish substance, very fusible, and possessing a peculiar and unpleasant odor. The quantity yielded by a pound of hepatic tissue is about five drachms and a half.

Frequently the surface of the liver presents small brownish spots, which, from the manner in which they are arranged, give it a very singularly mottled appearance. When a section is made, the interior is found to be of a much more uniform color, being of a pale yellow, deep orange, or light ochre. The hepatic tissue is generally diminished in specific gravity, though this occurrence is by no means constant. In two specimens examined by Mr. Bird, the weight was only 1,027, whereas in the same amount of healthy liver it was 1,062.

This fatty degeneration occurs almost exclusively in phthisical subjects. Louis found it in forty-four persons out of the one hundred and twenty-three who died of this disease under his care in the Charity Hospital at Paris. This writer even goes so far as to contend that this state of the liver is never seen, except in connexion with tubercles of the lungs or of some other organs. That this, however, is not necessarily, although it may be generally, the case, is abundantly disproved by the observations of other pathologists.

Various hypotheses have been offered with a view of accounting for this curious state of the liver; but the only explanation which seems to me to be at all tenable, is that which ascribes it to chronic inflammation, with venous engorgement, giving rise to an augmentation of the secretion of fatty matter, which, according to the well known experiments of Braconnet and Vauquelin, is naturally contained in the organ. This opinion derives support and illustration from what is observed in the inferior animals. In the goose and duck, an almost complete adipous transformation of the liver may be induced in the course of a few weeks, by subjecting them to inactivity, withholding the light, and cramming their stomachs with a paste made of barley-meal, mutton suet and coarse sugar, mixed with milk. This mode of fattening fowls has been pursued for many years in the neighborhood of London, and it has been found that if the repletion be kept up longer than a fortnight, so much structural lesion will be induced as to kill them, or render the meat unfit for the table, the liver being converted into a soft, red, oily mass. In the human subject, this change seems to take place very rapidly in some instances, as in the course of five or six weeks; and the organ is not unfrequently double the usual weight and bulk. Louis has ascertained that it is more common in young than in old persons, and in females than in men, in the ratio nearly of four to one.

The bile seems to be considerably altered in this affection, both in its chemical and physical properties. Dr. Addison of London, who has published an able essay upon the subject in a late number of Guy's Hospital Reports, states, that this fluid is frequently of a dirty brown color, and contains an infinite number of carbonaceous granules. After the addition of almost any of the acids, a most intolerable odor is evolved. The urine, too, is usually a good deal altered, being of very low specific gravity, quite neutral, and holding in suspension numerous flakes of mucus.

There are no signs, either general or local, which characterize this singular state of the liver. How far the symptom, recently pointed out by Dr. Addison, forms an exception to this remark, is a question which, for the present, must remain undecided. The symptom to which I allude, consists in a peculiar alteration of the color and texture of the integuments, most conspicuous on the face and back of the hands. These parts exhibit a singularly bloodless, almost semi-transparent, and waxy appearance, which by degrees becomes observable over the whole body. Sometimes the skin has a fine, polished, ivory look. To the touch, it is remarkably smooth, loose, flabby, and almost as soft as satin, apparently from the natural asperities being obliterated. In several instances, in which this peculiar condition of the integuments was present, has Dr. Addison confidently and correctly predicted the existence of the fatty degeneration of the liver. The subject is certainly of great interest, and will no doubt receive the serious attention of the profession.

29.—The *liver-fluke, the distoma hepaticum* of helminthologists, although said to have been several times found in the human subject, is much more common in the inferior animals, as the sheep, horse, stag and ox. It is also found in the gall-bladder, and Pallas mentions that he once detected it in the hepatic duct of a young female. Somewhat lanceolated in shape, it is of a yellowish color, obtuse at either extremity, and scarcely a fourth of an inch long, by one line in breadth. It has two openings, one in front, which is directed obliquely inwards, and another behind and inferiorly, which is slightly prominent, and answers to the anus; the neck is rounded, and of a light brownish hue; and the belly is marked by spots of an opaque dingy white. The insect is thought to have a distinct genital apparatus, with a vascular, and probably also a nervous system. In animals it is often an inch long, by nearly half an inch broad.

30.—*Cartilaginous* depositions are sufficiently rare in this organ; most frequently they are confined to the investing membranes, occurring in small patches, of a soft texture and greyish color. Dr. Roberts, of Paris, relates a case in which the whole of both tunics was converted into one mass of cartilage, in some places half an inch thick. In the midst of this substance were several scales of bone; one of them of the size of a half-crown piece.

31.—The depositions are sometimes entirely *osseous*. Dr. Venables met with a case in which the concretion, consisting almost entirely of phosphoric acid and lime, was about the size of the patella, with very much the same shape. It was embedded in the parenchymatous structure, and resembled common bone.* In other cases the earthy matter is soft, gristly, of a brownish white color, and enclosed in a dense, firm, fibro-cartilaginous cyst.

Very recently I have met with very small osseous concretions in several livers which were otherwise perfectly healthy. In one of the cases there were altogether about fifteen, varying in volume from a mustard seed to a currant. They were hard, somewhat gritty, of a yellowish hue, and most of them distinctly encysted, the capsule, which was of a greyish color, and fibro-cartilaginous firmness, being from the sixth to the fourth of a line in thickness, and closely adherent to the surrounding tissues. In

* London Cyclop. Pract. Med. vol. iv. p. 611.

another case, the concretions were only five or six in number, the largest of which was about the magnitude of a common pea. They were also encysted, but of a light brownish color, and of great hardness. How these little bodies originate, cannot be very easily determined. In one of the cases now referred to, it seemed to me that they were nothing but tubercles in a state of osseous degeneration; a supposition which derives strength from the fact that several of them, evidently less advanced than the rest, contained a minute quantity of curdy, friable matter. The individual, moreover, was of a decidedly scrofulous habit, and numerous tubercles were detected in different organs.

32.—Blood sometimes escapes into the substance of the liver, constituting the disease to which some of the French writers have applied the name of *hepatic apoplexy*. It generally proceeds from one or more ruptured vessels distributed through the parenchymatous texture, and occurs in small spots, varying in density and color according to the length of time they have existed, being always softer and darker the more recent they are. The disease, I presume, is extremely rare, as very few pathologists make mention of it. In my own dissections I have never met with an instance of it; nor am I aware that any of my friends have been more successful. No symptoms have yet been pointed out as indicative of hepatic apoplexy; nor is it reasonable to suppose that the organ would suffer much inconvenience unless the effusion should be copious.

33.—The liver is very easily *ruptured*, in consequence of external violence; and when this happens, large quantities of blood are liable to escape into the abdominal cavity. The accident is always fatal, the patient seldom surviving more than a few hours. To this statement, however, a remarkable exception is to be found in a case, the particulars of which have been recently given by Dr. Fazely of the Island of Trinidad. The individual, a man fifty years of age, survived the effects of the injury eight years, and finally died from a complication of disease. On examination, it was found that the right lobe of the liver had been ruptured throughout its whole length and substance, from the anterior to the posterior part, and that the lacerated edges had perfectly re-united through the medium of a broad, thick cicatrix, of a hard cartilaginous texture. In the interior of this cicatrix, at the depth of half an inch, a considerable cavity was seen, which communicated with the hepatic substance, and contained about fifty biliary concretions. The surface of the liver, it should be remarked, adhered extensively to the walls of the abdomen. The gall-bladder was empty and flaccid, and the alimentary tube perfectly sound.

That such accidents are not so fatal as has been generally imagined by the profession, may be inferred from some experiments performed by Professor Monro of Edinburgh. He opened the abdomen of a living rabbit, and cut off one of the lobes of the liver. The divided vessels bled very profusely; but by pressure, the hemorrhage entirely ceased in the course of three or four minutes. After the wound was sewed up, the animal appeared uneasy, but only for a short time, when it gradually recovered its spirits, and took food as before. During the five weeks that it was kept and closely watched by the experimenter, it was in perfect health, the digestion going on well, and the alvine evacuations being of the usual color, quantity, and consistence.

In another experiment, also performed upon a rabbit, the results were precisely similar. The animal lived upwards of twelve weeks, when it was killed. On examination, the cut surface of the liver was found firmly glued to the walls of the abdomen, by means of lymph.* The results of these experiments are extremely interesting, as showing how large a portion of an organ, so important to life, may be ablated or destroyed, and yet the animal perfectly recover.

With these remarks we close the present paper. Imperfectly as it has been treated, I yet hope that it will not be entirely without interest to the readers of this journal, for whose benefit it has been constructed. Nor shall I consider my time and labor as misspent, if what has been said shall have a tendency to arouse the attention of Western and Southern practitioners to the subject of the morbid anatomy of an organ, of which so much is talked but so little understood in this country. In the next number of the journal, I propose to take up the consideration of the various lesions of the gall-bladder and biliary ducts, with the pathology of jaundice.

---

* Elements of Anatomy, vol. i. p. 567. Edinb. 1831.

